# Validation of Assaying Carcinoembryonic Antigen in Human Serum by Using Immunomagnetic Reduction

**DOI:** 10.1038/s41598-018-28215-1

**Published:** 2018-07-03

**Authors:** Po-Li Wei, Long-Teng Lee, Li-Ming Tseng, Kai-Wen Huang

**Affiliations:** 1 Department of Surgery, College of Medicine, Taipei Medical University, Taipei, Taiwan; 2 Division of General Surgery, Department of Surgery, Taipei Medical University Hospital, Taipei Medical University, Taipei, Taiwan; 3 Cancer Center, Taipei Medical University Hospital, Taipei Medical University, Taipei, Taiwan; 4 Graduate Institute of Cancer Biology and Drug Discovery, Taipei Medical University, Taipei, Taiwan; 5 Department of Family Medicine, College of Medicine, National Taiwan University and Hospital, Taipei, 100 Taiwan; 6 Division of Colorectal Surgery, Department of Surgery, Far Eastern Memorial Hospital, New Taipei City, 220 Taiwan; 7 Department of Surgery and Hepatitis Research Center, National Taiwan University Hospital, College of Medicine, Taipei, 100 Taiwan; 8 Graduate Institute of Clinical Medicine, College of Medicine, National Taiwan University, Taipei, 100 Taiwan

## Abstract

Immunomagnetic reduction (IMR) is a method to assay biomolecules by utilizing antibody functionalized magnetic nanoparticles. For clinical validation, important analytic performances of assaying carcinoembryonic antigen (CEA) using IMR are characterized. Furthermore, IMR is applied to assay carcinoembryonic antigen (CEA) in human serum for clinical validation. A total of 118 healthy controls and 79 patients with colorectal cancer (CRC) are recruited in this study. For comparison, assays using chemiluminometric immunoassay (CLIA) are also done for quantizing CEA in these serum samples. The results reveal a high correlation in terms of serum CEA concentration detected via IMR and CLIA is found (r = 0.963). However, IMR shows higher clinical sensitivity and specificity than those of CLIA. Moreover, the rate of false positives for smoking subjects is clearly reduced through the use of IMR. All the results demonstrate IMR is a promising alternative assay for serum CEA to diagnose CRC.

## Introduction

Colorectal cancer (CRC) is the third common cancer in men and in women. More than 130,000 new cases of CRC are diagnosed each year^1,2^. Fortunately, the 5-year survival rate after treatment for early-stage CRC patients is higher than 60%^3–5^. Thus, screening tests for early-stage diagnosis of CRC have become important and also promoted in many countries^6–10^. Many reports point out screening tests for CRC patients reduce colorectal-cancer mortality by 50%^11,12^. The most frequently used test is to assay carcinoembryonic antigen (CEA) in human serum^13–15^. A large number of commercially available products utilizing different technologies, such as sandwiched enzyme-linked immunosorbent assay (ELISA)^16,17^, immunonephelometry^18,19^, and chemiluminometric immunoassay (CLIA) etc.^20,21^, have been widely applied in clinics. However, there are several problems with assaying CEA in human serum using these assays. For example, it is not easy to avoid interference caused by hemoglobin, bilirubin, lipid, and chemical drugs in the serum^22^. Thus, the diagnostic accuracy of CRC is seriously challenged by assaying serum CEA. In practice, the clinical sensitivity and specificity of diagnosing CRC via serum-CEA assay is 60–70%^23–25^. In particular, the occurrence rate of false positives is extremely high for the smoking population^26,27^. It is therefore truly necessary to develop an alternative method to assay serum CEA with higher accuracy for diagnosing CRC.

In 2006, the so-called immunomagnetic reduction (IMR) method was proposed^28^. In IMR, antibody functionalized magnetic nanoparticles dispersed in PBS solution act as a reagent. Under external alternative-current (AC) magnetic fields, magnetic nanoparticles are oscillated and an AC magnetic signal is generated with the reagent. Once magnetic nanoparticles associate with target biomolecules, the effective mass of bound magnetic nanoparticles increases, resulting in the suppression of the oscillating efficiency of the magnetic nanoparticles^29^. Consequently, the AC magnetic signal of the reagent is reduced. The reduction in the AC magnetic signal of the reagent increases logistically with the increasing concentration of target biomolecules^30^. Since IMR is a homogeneous assay and the binding area of magnetic nanoparticles with target biomolecules is very large, the sensitivity of IMR is ultra-high. Many published papers demonstrate ultra-high sensitivity in assaying protein, virus, and chemicals via IMR^31–33^. Besides, the interference for assaying target biomolecules can be suppressed in IMR, as evidenced in refs^22,34–37^. With its ultra-high sensitivity and specificity, IMR is a promising candidate to achieve accurate *in-vitro* diagnosis. One of impacts attributed from high-sensitivity and high-specificity assay is early-stage diagnosis in clinics. Early-stage diagnosis can help medical doctors to treat patients timely and adequatly. Thus, not only the medical cost but also the mortality can be significantly reduced.

In our previous study^37^, some analytical performances, such as reagent stability, interference tests, and assay linearity, of assaying CEA using IMR were investigated. The results reveal the promising feasibility of using IMR for quantitatively detecting CEA in human serum for clinical application. However, there are several analytical performances of assaying CEA using IMR unclear, including Hook effect, limit of background, limit of detection, dilution recovery range, precision, and reproducibility of assay, etc. Moreover, it lacks strong evidence to validate its clinical performance. Hence the reported IMR CEA assay is not ready for clinical use. Completed investigations on analytical performce and well-designed clinical trails are necessary to validate the clinical significance of assaying serum CEA using IMR. In this work, in addition to investigating analytical performances, IMR is applied to assay CEA in the human serum of 118 healthy controls and 79 patients with CRC. The quality management of this clinical study follows the guildlines of Good Clinical Practice. The design of the validation for clinical use of IMR CEA assay follows 510k guildlines. Thus, all serum samples have to be assayed with CEA using clinically approved technology, such as chemiluminometric immunoassay (CLIA). The correlation in terms of detected serum CEA concentration between IMR and CLIA is explored. Moreover, the clinical sensitivity and specificity for diagnosing CRC via assaying serum CEA by using IMR and CLIA are characterized. To our knowledge, the current work is the first one to formally validate the clinical significance of IMR technology.

## Material and Methods

### Hook Effect of Assay

10 μl of purified human CEA protein (1 mg/ml; Cat. No.PHP282; AbDSerotec) was spiked into a 9,990 μl of serum pool to reach a stock of 10 μg/ml (=10,000 ng/ml) CEA-serum sample, followed by using the stock to mix with serum by various volume ratio to get 10 CEA-serum samples containing 0.1, 0.5, 1.0, 5.0, 10, 25, 50, 100, 300, 600 and 1,000 ng/ml of human CEA protein. Then 40-μl of reagent (MF-CEA-0061, MagQu) is mixed well with 60-μl of CEA-serum sample in the sample assaying tube and put into the assaying channel of IMR analyzer (XacPro-E, MagQu) for reading IMR signals. Thus, the CEA concentration dependent IMR signal is established to investigate Hook effect.

### Limit of Background and Detection of Assay

The processes for examining limit of background (LoB) and limit of detection (LoD) are following the guidelines CLSI EP17-A. The sample for LoB test is phosphate buffered saline (PBS) (pH 7.4), while 0.5-ng/ml CEA-serum sample is used for LoD test. According to CLSI EP17-A, the numbers of tests for either LoB or LoD are prepared for at least 60. For each test, duplicated measurements of CEA concentration via IMR are performed.

### Dilution Recovery of Assay

For studying dilatation recovery range, CEA-serum sample with known purified human CEA concentration, say 90.47 ng/ml, was diluted by PBS buffer with factors of 2, 4, 8, 16, 32 and 160. The CEA concentration for each diluted sample is measured via IMR with duplicated measurements. The dilution recovery was determined by the ratio between the measured CEA concentration and expected CEA concentration, as expressed

1 $${\rm{Dilution}}\,{\rm{recovery}}=\frac{{\rm{Measured}}\,{\rm{concentration}}}{{\rm{Expected}}\,{\rm{concentration}}}\times 100 \% ,$$

where the measured concentration is the mean value of duplicated-measured CEA concentrations.

### Precision and Reproducibility of Assay

The precision and reproducibility testing was conducted in accordance with the CLSI/NCCLS Approved Guideline for Evaluation of Precision Performance of Quantitative Measurement Methods, EP5-A2. The CEA-serum samples were measured in duplicate, once per day over 20 days. The two measurements on two sequent days are regarded as two runs. Two serum pools (serum pool 1 and serum pool 2) with different and unknown CEA concentration were used.

### Collection of Human Serum

In this study, 275 subjects were recruited from December 1, 2014 to June 30, 2015 at three hospitals in Taiwan. The inclusion criteria for healthy controls and CRC patients were over 20 years of age, male or female, and a body weight greater than 50 kg. All study subjects provided informed consent prior to participation in this investigation, and the study was approved by the ethics committee and the institute review board (IRB) of National Taiwan University Hospital (IRB No. 201403089DSC), Taipei Medical University Hospital (IRB No. 201406006), and Far Eastern Memorial Hospital (IRB No. FEMH-1-103140-F). All experiments were performed in accordance with relevant guidelines and regulations.

For each subject, 8 ml of whole blood was collected in an anticoagulant-free serum separation tube (red top). Serum was separated from blood cells by centrifuging the whole blood sample at 1,500–2,000 × g for 10 minutes. Following centrifugation, serum was taken by pippetman and equally divided into four 1.5 ml microcentrifuge tubes (1 ml each). Serum samples were labeled and deep frozen (−20 °C or colder) until further use.

### Comparison Study of IMR Assay

To assess the correlation and compare the accuracy between IMR and CLIA, the serum CEA concentration of a subject was measured simultaneously using two commercial assay kits: SIEMENS Reagents for CEA assay and MagQu IMR CEA assay. The details of the operating procedures for assaying CEA using CLIA and IMR were performed according to the manuals of the two assay kits. The measuring ranges of CEA using SIEMENS Reagents for the CEA assay and MagQu IMR CEA assay were from 0.5 to 100 ng/ml and from 0.21 to 100 ng/ml, respectively. Samples showing CEA concentrations within the measuring ranges were valid. Among the recruited 275 subjects, 197 subjects devoted valid serum samples. The demographic information of the 197 subjects is listed in Table 1. 118 subjects were healthy controls and the other 79 subjects were CRC patients diagnosed with pathological evidence. The 197 healthy controls included 46 men and 72 women. The 79 CRC patients included 43 male subjects and 36 female subjects. The average age of the healthy controls was (44.6 ± 16.4) years, while the CRC patients were aged (62.0 ± 11.5) years. The body weights of the healthy controls and CRC patients were (63.9 ± 10.5) kg and (63.7 ± 10.9) kg, respectively. Seven of the 118 healthy controls and 11 of the 79 CRC patients were frequent smokers.

**Table 1:** Demographic information of 197 subjects validly recruited in this study.

Group	Healthy controls	CRC patients
Nos. of subjects	118	79
Gender (Meal/Female)	46/72	43/36
Age (years)	44.6 ± 16.4	62.0 ± 11.5
Body weight (Kg)	63.9 ± 10.5	63.7 ± 10.9
Nos. of smoker	7	11

The correlation and similarity between the serum CEA concentrations of subjects assayed with CLIA and IMR were determined by Pearson correlation coefficient and regression analysis.

## Results

### Analytic Performance

**Figure 1:**
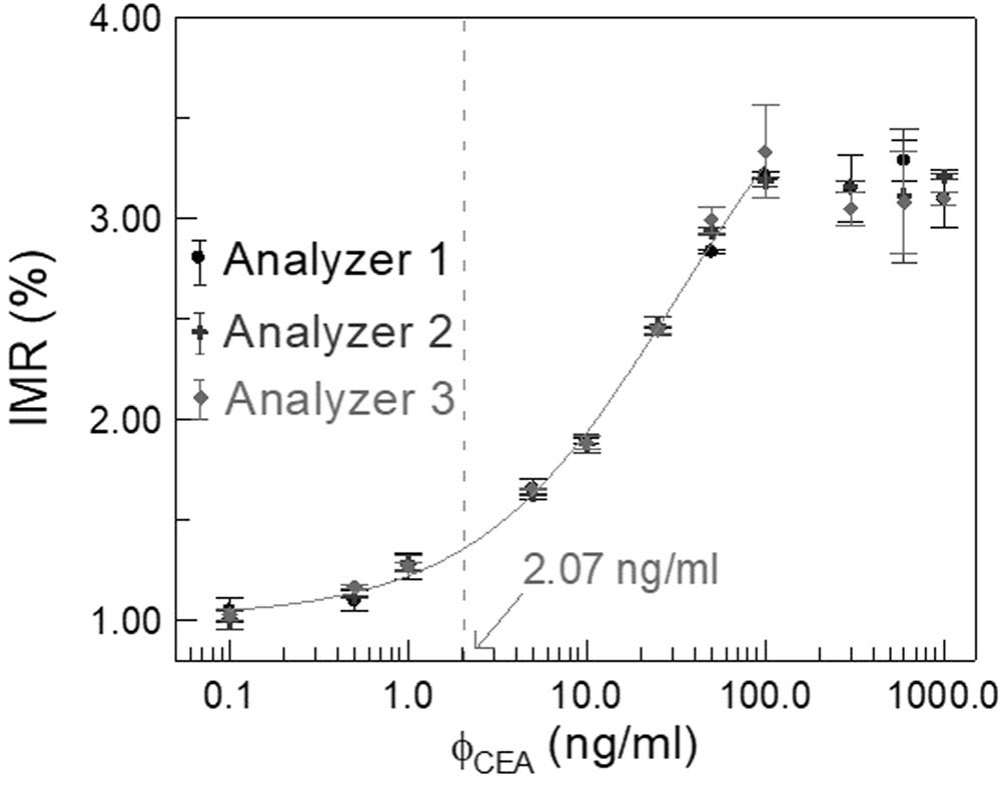
Relationship between IMR signal and the CEA concentration in human serum. The cut-off value of serum CEA concentration to discriminate CRC from healthy subjects is 2.07 ng/ml and is labled with the dashed line.

CEA-serum samples of various concentrations from 0.1 ng/ml to 1000 ng/ml are used for the IMR measurements to establish the relationship between IMR signal and CEA concentration. The measured IMR signals for CEA-serum samples are shown in Fig. 1. The error bar with each data point in Fig. 1 attributes from the duplicate measurements. The mean value in IMR signal of the duplicate measurements is denoted with the data point. Since three analyzers were used, there are three data points for a certain CEA-serum sample. The averaged IMR signals are used for establishing the analytic relationship between IMR signal and CEA concentration in serum.

It is clear in Fig. 1 that the IMR signal increases as CEA concentration increases. However, as the CEA concentration is higher than 100 ng/ml, the IMR signal slightly decreases. The decrease in IMR signal for CEA concentration higher than 100 ng/ml is resulted from Hook Effect. Hence, the averaged IMR signals, IMR(%) for CEA concentrations, ϕ_CEA_, from 0.1 ng/ml to 100 ng/ml are used for exploring the analytic relationship, which follows the logistic function

2 $$IMR( \% )=\frac{A-B}{1+{(\frac{{\varphi }_{CEA}}{\varphi })}^{\gamma }}+B,$$

where A, B, γ and ϕ_o_ are fitting parameters. By fitting the CEA concentration dependent averaged IMR signals to Eq. (2), the parameters are found to be A = 1.01, B = 4.25, γ = 0.76, ϕ_o_ = 33.35, and its coefficient of determination R^2^ is 0.998. The fitted logistic function is plotted with the solid line in Fig. 1.

According to the CLSI EP17-A, the limit of background (LoB) is established as followed. The measurements are ordered according to their values, and the appropriate percentile (p) is estimated as the value of the observation with rank value as determined below; in this case, p = 0.95:

3 $${\rm{LoB}}={\rm{Results}}\,{\rm{at}}\,{\rm{position}}\,[0.95\times {\rm{NB}}+0.5],$$

where NB is the number of repeating testing (=60 in this work). Equation (18.2) becomes

4 $${\rm{LoB}}={\rm{Results}}\,{\rm{at}}\,{\rm{position}}\,57.5$$

This is a noninteger value. Meanwhile, the distribution of 60 testing results shows non-Guassian distribution. Linear interpolation is carried out using the 57^th^ and 58^th^ ranked observations according to CLSI EP17-A. The 60 measured concentrations for serum samples without being spiked with CEA, i.e. blank samples, are ranked in Table 2. By using the 57^th^ and 58^th^ ranked observations for the linear interpolation, the 57.5^th^, which denotes the mean measured concentration of 57^th^ and 58^th^ test, the measured concentration is calculated as 0.09 ng/ml, which is the limit of background for assaying CEA using IMR.

**Table 2:** Ranking list of the 60 measured concentrations for serum samples without being spiked with CEA.

Rank	Measured concentration (ng/ml)	Rank	Measured concentration (ng/ml)	Rank	Measured concentration (ng/ml)
1	−1.25	21	−0.57	41	−0.22
2	−1.23	22	−0.57	42	−0.20
3	−1.21	23	−0.55	43	−0.20
4	−1.17	24	−0.53	44	−0.18
5	−1.06	25	−0.51	45	−0.17
6	−1.06	26	−0.50	46	−0.17
7	−1.05	27	−0.48	47	−0.17
8	−0.99	28	−0.48	48	−0.15
9	−0.97	29	−0.46	49	−0.13
10	−0.86	30	−0.42	50	−0.11
11	−0.86	31	−0.40	51	−0.09
12	−0.81	32	−0.40	52	0
13	−0.79	33	−0.39	53	0
14	−0.73	34	−0.37	54	0
15	−0.68	35	−0.35	55	0.03
16	−0.68	36	−0.35	56	0.07
17	−0.68	37	−0.31	57	0.09
18	−0.66	38	−0.31	58	0.09
19	−0.61	39	−0.26	59	0.11
20	−0.61	40	−0.26	60	0.16

According to the CLSI EP17-A, the limit of detection (LoD) is calculated via

5 $${\rm{LoD}}={\rm{LoB}}+1.645\,{{\rm{\sigma }}}_{{\rm{S}}},$$

where σ_S_ is the standard deviation of the measured CEA concentrations of CEA-serum samples at a given spiked CEA concentration, say 0.5 ng/ml in this work. The CEA concentrations of 60 CEA-serum samples were measured using the reagent MF-CEA-0061 and the analyzer XacPro-E. For each sample, duplicate measurements were done. The mean value of the duplicate measurement for each sample is listed in Table 3.

**Table 3:** List of the 60 measured concentrations for serum samples spiked with 0.5-ng/ml CEA.

Rank	Measured concentration (ng/ml)	Rank	Measured concentration (ng/ml)	Rank	Measured concentration (ng/ml)
1	0.44	21	0.68	41	0.68
2	0.47	22	0.57	42	0.53
3	0.51	23	0.64	43	0.66
4	0.44	24	0.66	44	0.69
5	0.57	25	0.49	45	0.60
6	0.53	26	0.49	46	0.57
7	0.66	27	0.62	47	0.51
8	0.60	28	0.58	48	0.62
9	0.66	29	0.47	49	0.49
10	0.55	30	0.62	50	0.53
11	0.53	31	0.55	51	0.69
12	0.60	32	0.68	52	0.53
13	0.55	33	0.68	53	0.53
14	0.55	34	0.49	54	0.47
15	0.47	35	0.58	55	0.64
16	0.49	36	0.66	56	0.60
17	0.57	37	0.51	57	0.66
18	0.53	38	0.47	58	0.64
19	0.51	39	0.53	59	0.60
20	0.51	40	0.60	60	0.51

The mean measured of above 60 measured concentrations is 0.57 ng/ml. The standard deviation σ_S_ of above 60 measured concentrations is 0.07 ng/ml. The LoD is obtained as 0.21 ng/ml via Eq. (5).

A dilution recovery study was performed by diluting a serum sample by factors of 2, 4, 8, 16, 32 and 160. The measured CEA concentration of the original serum sample (un-diluted) is 90.47 ng/ml. The expected concentration and measured concentration for diluted serum samples are listed in Table 4. The dilution recovery for each diluting factor is also listed. The recoveries for samples diluted 1:2, 1:4, 1:8, 1:16, and 1:32 ranged from 94.7% to 109.7% with a mean of 102.5%, while the recovery for samples diluted 1:160 is out of the range from 90% to 110%. So, the highest dilution factor is 32 times.

**Table 4:** Dilution factors, expected concentration, measured concentration, and dilution recovery for diluted serum samples.

Dilution factor	Expected concentration (mean; ng/ml)	Measured concentration (mean; ng/ml)	Dilution recovery
2	45.24	46.69	103.2%
4	22.62	24.50	108.3%
8	11.31	10.72	94.7%
16	5.65	6.20	109.7%
32	2.83	2.74	96.8%
160	0.57	0.80	140.4%

Two pools of serum samples were used for precision and reproducibility tests. The measured CEA concentrations using IMR reagent and analyzer are listed in Table 5 for serum pool 1 and in Table 6 for serum pool 2. The mean concentration of each pool are 10.08 ng/ml (pool 1) and 2.22 ng/ml (pool 2), respectively. According to the statistical method mentioned in CLSI/NCCLS Approved Guideline for Evaluation of Precision Performance of Quantitative Measurement Methods, EP5-A2, the statistical results comes from Tables 5 and 6 showed the standard deviations of repeatability and within-lab for various CEA concentrations are shown in Table 7. The imprecision (%CV) was less than 15%.

**Table 5:** Measured CEA concentrations in serum pool 1 sample for the analysis of precision and reproducibility.

Measurement day	Measured CEA concentrations (ng/ml) in duplication	
1	9.12	11.55
2	7.20	8.57
3	9.69	12.00
4	8.93	8.39
5	11.12	10.08
6	10.08	8.93
7	10.91	8.93
8	8.39	8.57
9	9.89	13.41
10	12.46	11.12
11	11.77	10.29
12	11.77	10.29
13	9.89	8.57
14	9.89	9.50
15	9.30	10.08
16	10.29	10.49
17	10.29	11.55
18	9.12	7.04
19	10.91	10.29
20	10.91	11.55

**Table 6:** Measured CEA concentrations in serum pool 2 sample for the analysis of precision and reproducibility.

Measurement day	Measured CEA concentrations (ng/ml) in duplication	
1	2.63	2.44
2	1.99	1.65
3	1.82	1.95
4	2.25	1.90
5	2.34	1.99
6	2.53	1.99
7	2.07	1.65
8	1.99	2.44
9	2.53	2.07
10	2.34	2.82
11	2.25	2.34
12	1.99	2.16
13	2.07	1.82
14	2.16	2.16
15	2.16	1.90
16	2.34	2.25
17	2.34	1.90
18	2.82	2.53
19	2.44	2.53
20	2.44	2.92

**Table 7:** Standard deviations of repeatability and within-lab for assaying CEA concentrations in serum samples using IMR reagent and analyzer. The unit for CEA concentration in ng/ml.

Sample	Mean of measured CEA concentrations	Standard deviation (%CV)	
		Repeatability	Within-Lab
Serum pool 1	10.08	1.09 (10.8)	1.39 (13.8)
Serum pool 2	2.22	0.24 (10.8)	0.31 (14.0)

### Clinical Studies

**Figure 2:**
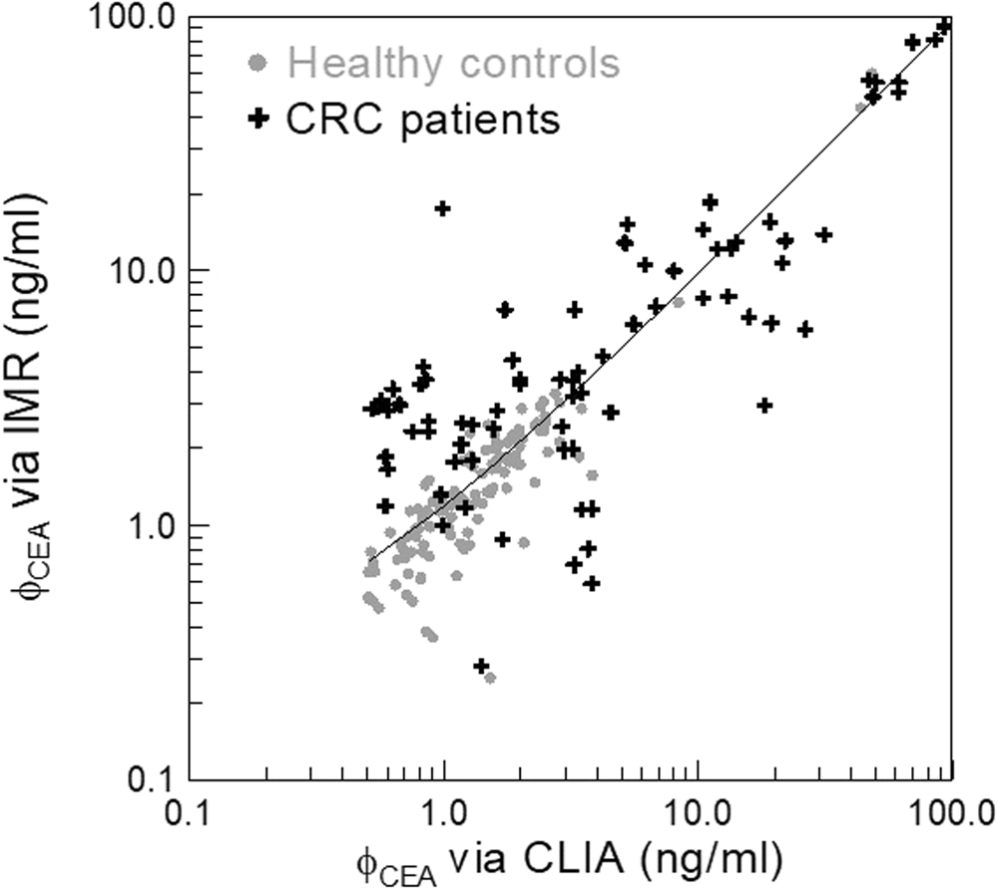
Detected CEA concentrations in human serum by using CLIA (x axis) and IMR (y axis).

The CEA concentrations in the human serum samples assayed using CLIA and IMR are shown in Fig. 2. The gray dots denote the healthy controls, while the black cross symbols stand for the CRC patients. Irrespective of healthy controls or CRC patients, all data points in Fig. 2 span from the lower-left to the upper-right. This reveals the CEA concentrations detected using IMR are linear to those assayed using CLIA. The Pearson correlation coefficient between the CEA concentrations assayed by IMR and CLIA were found to be 0.963. A high linear correlation in CEA concentrations is demonstrated between IMR and CLIA. The linear relationship is shown by the solid line in Fig. 2 and can be expressed as

6 $${\varphi }_{{\rm{CEA}},{\rm{IMR}}}=0.931\times {\varphi }_{{\rm{CEA}},{\rm{CLIA}}}+0.273,$$

where ϕ_CEA,IMR_ and ϕ_CEA,CLIA_ are the detected CEA concentrations using IMR and CLIA, respectively. The slope in Eq. (6) lies in the range from 0.9 to 1.1, which matches the FDA 510k regulation.

**Figure 3:**
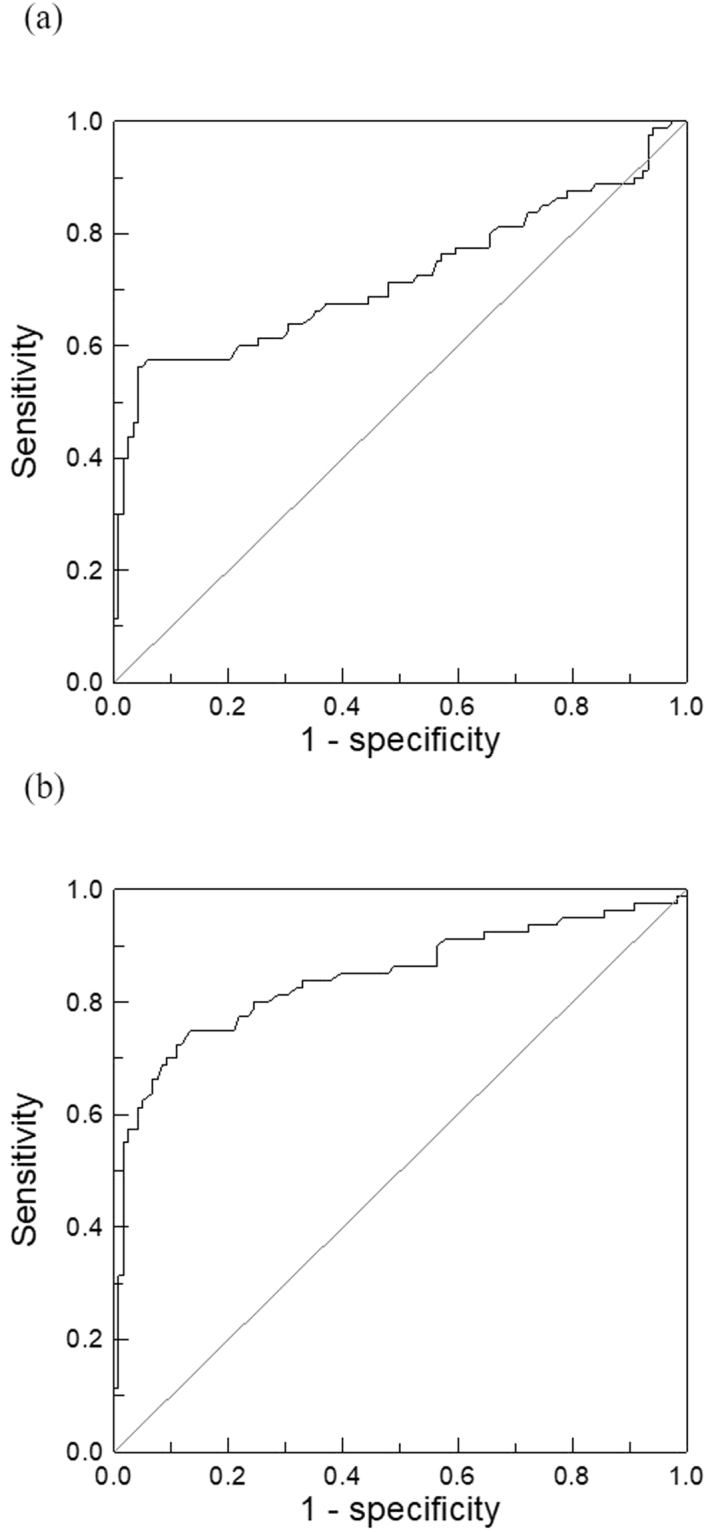
ROC curves for diagnosing CRC via assaying serum CEA by using (**a**) CLIA and (**b**) IMR.

In Fig. 2, the gray dots distribute in the lower-left area of Fig. 2. The black cross symbols extend to the upper-right area of Fig. 2. This implies healthy controls show lower CEA concentrations in the serum compared with those of the CRC patients. The clinical sensitivity and specificity of diagnosing CRC via serum CEA concentrations using CLIA and IMR are analyzed via the receiver operating characteristic (ROC) curve, respectively. The ROC curve for CLIA is shown in Fig. 3(a). The cut-off value for diagnosing CRC via the serum CEA concentration using CLIA was found to be 1.69 ng/ml, with clinical sensitivity and specificity being 0.646 and 0.695. The area under the ROC curve in Fig. 3(a) is 0.713.

As for IMR, the ROC curve for diagnosing CRC according to serum CEA concentration is shown in Fig. 3(b). The cut-off value was found to be 2.07 ng/ml, showing clinical sensitivity and specificity of 0.785 and 0.780 for diagnosing CRC via serum CEA concentration using IMR. The area under the ROC curve in Fig. 3(b) is 0.845. Clearly, the diagnostic accuracy of CRC in terms of serum CEA concentration is increased by more than 10% using IMR instead of CLIA.

**Figure 4:**
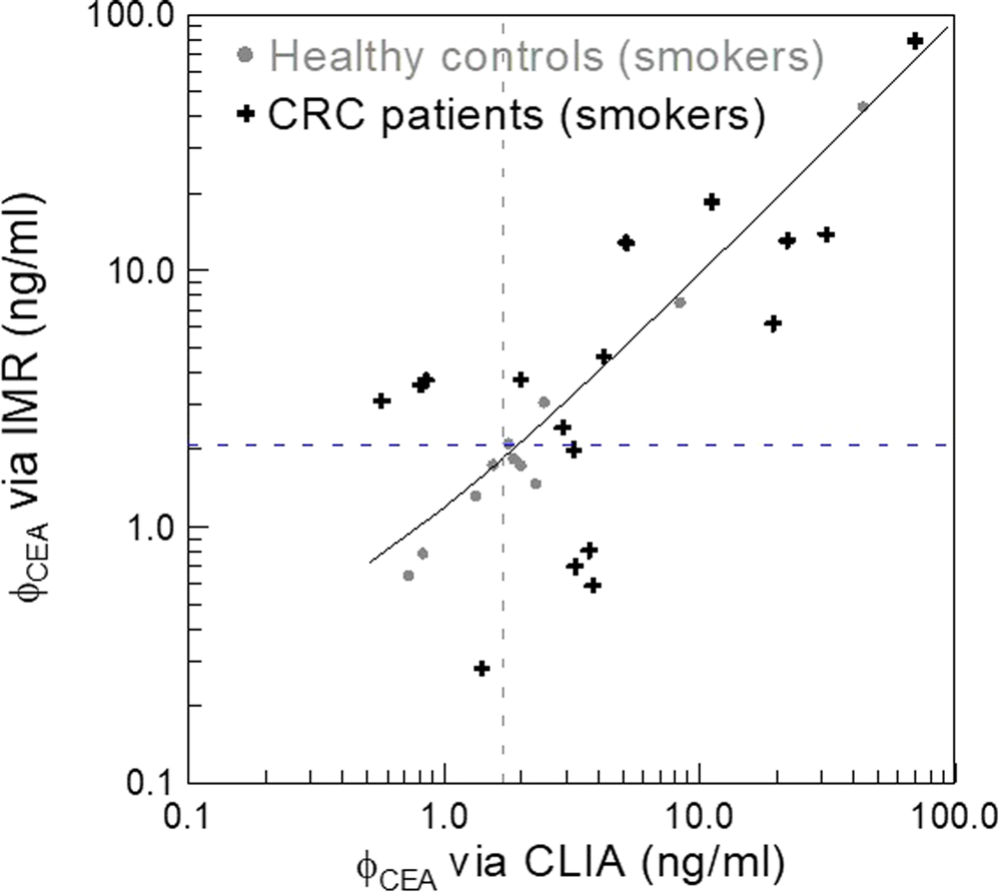
Detected CEA concentrations in human serum of smoking subjects by using CLIA (x axis) and IMR (y axis). The dashed lines are the cut-off values for diagnosing CRC obtained through the analysis of ROC curves in Fig. 2.

It is well known the false positive rate for diagnosing CRC in the smoking population is extremely high. For example, as shown in Fig. 4, by applying the cut-off value with CLIA, i.e. 1.69 ng/ml plotted with the dashed vertical line, to smoking subjects, seven of eleven smoking healthy controls are identified as positive (CRC patients). This results in a clinical specificity of 0.364 for diagnosing CRC using CLIA. The corresponding clinical sensitivity is 0.765. As for IMR, by applying the cut-off value, i.e. 2.07 ng/ml plotted with the dashed horizontal line in Fig. 4, only four of eleven smoking healthy controls are identified as CRC patients. The clinical specificity is 0.636 and the sensitivity is 0.706. Remarkably, the false positive for diagnosing CRC via assaying serum CEA by using IMR can be significantly suppressed as compared with CLIA. The characteristics will be very useful because the false positive result of CEA in smokers always confuses the clinician and results in medical resource wastage.

The reasons for IMR showing higher accuracy in diagnosing CRC by assaying serum CEA might be due to its high specificity. To investigate the specificity, the interference effect contributed by commonly existing molecules in serum for assaying CEA is examined. All the interfering materials, including nicotine, and their tested concentrations are listed in Table 8. Note, the concentrations of these interfering materials are much higher than normal levels in human blood. Each sample is spiked with both 5-ng/ml CEA and one kind of interfering material. The sample No. 1 is pure 5-ng/ml CEA solution, without any interfering material, and is used as a reference sample. The CEA concentrations of all samples are detected via IMR. The ratio of the detected CEA concentration of any sample with the interfering material to that of reference sample is defined as the recovery rate. As listed in Table 8, the recovery rate for each sample with interfering material exists within the range from 90% to 110%, revealing the interference effect of common molecules in blood, including nicotine, when assaying serum CEA can be ignored by using IMR. In measuring the serum CEA level, the confounding effect of smoking seems to be alleviated by the new method, however, further study with more enrolled smokers will be necessary.

**Table 8:** Recovery rates for assaying CEA in human serum with interfering material by using IMR.

Sample No.	Interfering material/Concentration	CEA concentration (ng/ml)	Recovery rate (%)
1	None	5.42	—
2	Hemoglobin/10000 μg/ml	5.15	95.0
3	Conjugated bilirubin/600 μg/ml	5.28	97.4
4	Intra lipid/30000 μg/ml	5.28	97.4
5	Uric acid/200 μg/ml	5.15	95.0
6	Rheumatoid factor/500 IU/ml	5.88	108.5
7	Albumin/60000 μg/ml	5.30	97.8
8	Acetylsalicylic acid/500 μg/ml	5.42	100.0
9	Ascorbic acid/300 μg/ml	5.76	106.3
10	Tegafur with uracil/50 μg/ml	5.77	106.5
11	Furosemide/4000 μg/ml	5.49	101.3
12	Levodopa/20 μg/ml	5.15	95.0
13	Ampicillin sodium/1000 μg/ml	5.28	97.4
14	Nicotine/100 ng/ml	5.62	103.7

The mechanisms to achieve the high specificity for IMR have been discussed in ref.^38^. Briefly, there are two main reasons for the high specificity of IMR. The first is IMR detects the magnetic signal instead of the optical signal. Thus, the color interference, which usually occurs due to the existence of hemoglobin, bilirubin, or lipid in serum and induces significant variations in optical signals, vanishes in IMR. The second cause is the suppression of non-specific binding between the molecules and magnetic nanoparticles. Once the molecule associates with the antibody immobilized on a magnetic nanoparticle, the molecule is acted by a centrifugal force because the magnetic nanoparticle is oscillating under IMR measurement. The centrifugal force becomes strong as the oscillation frequency increases. Whenever the centrifugal force is stronger than the binding force between the molecule and antibody at higher oscillating frequencies, the association between the molecule and antibody is broken. The binding force between the specific molecule and antibody is stronger than that of non-specific molecules. This implies the centrifugal force can be adjusted to be between the binding forces of specific binding and non-specific binding. Thus, non-specific binding is eliminated and high specificity is the result. According to the published papers, IMR shows high assay specificity not only for CEA but also for many other kinds of proteins, such as alpha-fetoprotein^22^, β-amyloid^35^, c-reactive protein^36^, vascular endothelial growth factor^39^, desgammacarboxy prothrombin^40^, etc.

## Conclusion

By using IMR to assay CEA in human serum, the Hook effect occurs when the CEA concentration is higher than 100 ng/ml. The limit of background and detection are 0.09 ng/ml and 0.21 ng/ml, respectively. The highest dilution factor for CEA assay using IMR is 32 times. The imprecision of IMR for assaying CEA in human serum in is less than 15%. The results using IMR to quantitatively detect the concentrations of CEA in human serum are highly correlated (r = 0.963) to those using chemiluminometric immunoassay (CLIA). However, by using pathological evidence as a reference, the accuracy of diagnosing colorectal cancer according to serum CEA is elevated by 10% when using IMR as compared to CLIA. In particular, false positives are significantly suppressed in smokers by using IMR instead of CLIA. These results reveal IMR application in the clinical diagnosis of colorectal cancer by assaying serum CEA holds significant promise.
